# MODIFIED ALVARADO SCORE IN CHILDREN WITH DIAGNOSIS OF APPENDICITIS

**DOI:** 10.1590/0102-6720201700010014

**Published:** 2017

**Authors:** Mehran PEYVASTEH, Shahnam ASKARPOUR, Hazhir JAVAHERIZADEH, Sepideh BESHARATI

**Affiliations:** 1 Department of Pediatric Surgery, Imam Khomeini Hospital; 2 Department of Pediatric Gastroenterology, Abuzar Children's Hospital, Ahvaz Jundishapur University of Medical Sciences, Ahvaz, Iran

**Keywords:** Appendicitis, Nausea, Appendectomy.

## Abstract

**Background:**

Appendicitis is one of the most common abdominal emergency. Some predictive scoring systems are recommended to decrease the rate of negative appendectomy.

**Aim:**

To evaluate sensitivity, specificity, positive predictive value, and negative predictive value of modified Alvarado score in children who underwent appendectomy.

**Methods:**

Four hundred children with initial diagnosis of appendicitis were randomly selected from patients who underwent appendectomy. Modified Alvarado score was used for evaluation of the appendicitis, that was confirmed using histology.

**Results:**

Of modified Alvarado score components, anorexia; nausea and vomiting and rebound tenderness were significantly more common in children with positive appendectomy in contrast to patients with negative appendectomy. Sensitivity, specificity, positive predictive value, and negative predictive value for modified Alvarado score were: 91.3%; 38.4%; 87.7%; and 51.2% respectively.

**Conclusion:**

Alvarado score has high sensitivity but low specificity for diagnosis of acute appendicitis in children.

## RESUMO

**Racional:**

A apendicite é uma das emergências abdominais mais comuns. Alguns sistemas de pontuação preditivos são recomendados para diminuir a taxa de apendicectomia negativa.

**Objetivo:**

Avaliar a sensibilidade, especificidade, valor preditivo positivo e valor preditivo negativo do escore de Alvarado modificado em crianças submetidas à apendicectomia.

**Métodos:**

Quatrocentos crianças com diagnóstico inicial de apendicite foram selecionadas aleatoriamente de pacientes submetidos à apendicectomia. A pontuação de Alvarado modificada foi utilizada para avaliação do quadro, que foi confirmado por meio de histologia.

**Resultados:**

Anorexia; náuseas, vômitos e desconforto abdominal foram significativamente mais comuns em crianças com apendicectomia positiva, em contraste com casos negativos pelo escore de Alvarado modificado. A sensibilidade, especificidade, valor preditivo positivo e valor preditivo negativo para o escore de Alvarado modificado foram: 91,3%; 38,4%; 87,7%; e 51,2%, respectivamente.

**Conclusão:**

O escore de Alvarado possui alta sensibilidade, mas baixa especificidade para o diagnóstico de apendicite aguda em crianças.

## INTRODUCTION

Appendicitis is one of the important surgical emergency among children. Both pediatrician and surgeons should be aware the possibility of appendicitis in children with appendicitis. Despite its high incidence, diagnosis is difficult due to non specific symptoms and atypical presentations^3^. 

Variety of different approaches are recommended to decrease negative appendectomies such as predictive scoring system^2^, computer aided diagnosis, inflammatory marker^10^, ultrasonography^8^, and computed tomography.

Alvarado described a clinical scoring system on the basis of eight predictive clinical factors to improve the accuracy of physicians' clinical assessments in diagnosing acute appendicitis. This scoring system produces a maximum total score of 10 points and includes clinical symptoms (nausea and anorexia), signs( fever, shifting pain, right lower quadrant pain, and rebound tenderness) and laboratory findings( leukocytosis and neutrophilia). Right lower quadrant pain and leukocytosis contribute 2 points while the rest contributes 1 point^2^. Kalan et al. omitted shift to left parameter because is not routinely available in many laboratories, and produced a modified score^4^. The modified Alvarado score (MAS) has been widely accepted after it was successfully tested in different studies^1^. 

The aim of this study was to evaluate sensitivity, specificity, positive predictive value, and negative predictive value of modified Alvarado score in children who underwent appendectomy.

## METHODS

This study was approved by research affairs of Ahvaz Jundishapur University of Medical Sciences. This cross sectional study was carried out in Imam Khomeini Hospital, Ahvaz-Iran. It included 400 children with diagnosis of appendicitis during 2006-2012. Of children who underwent appendectomy 400 children were randomly selected. Appendicitis was confirmed according to pathology report. Modified Alvarado score was used to evaluate appendicitis^4^. Modified Alvarado score is based on three symptoms, three signs, and one laboratory investigation and ranged from 1-9 (Table 1)

**TABLE 1:** Modified Alvarado score

Migratory right iliac fossa pain	1
Anorexia	1
Nausea and vomiting	1
Tenderness of right lower quadrant	2
Rebound tenderness of right iliac fossa	1
Elevated temperature	1
Leukocytosis	2
Modified Alvarado Score	9

Appendicitis was confirmed using histopathology evaluation.

## RESULTS

In this study, 400 children aged <12 years with primary diagnosis of appendicitis were included. Of these cases, 337(84.3%) had confirmed appendicitis. Of all cases, 63(15.8%) had negative appendectomy. As seen in Table 2, anorexia, nausea and vomiting, and rebound tenderness were significantly more common in children with appendicitis than children without appendicitis. Migratory right iliac fossa pain was the most sensitive part of MAS (Table 3). Of 48 children with score 1-4, 45 had negative histopathology (Table 4). As seen in Table 4, all children with score 7-9 had positive histopathology. 

**TABLE 2:** Signs and symptoms of modified Alvarado score in the sample

		Appendicitis(+)	Appendicitis(-)	P value
Shifting pain	Yes	218(64.7%)	15(23.8%)	0.1
	No	119(35.3%)	48(76.2%)	
Anorexia	Yes	248(73.6%)	32(50.8%)	<0.001
	No	89(26.4%)	31(49.2%)	
Nausea and vomiting	Yes	284(84.3%)	38(60.3%)	<0.001
	No	53(15.7%)	25(39.7%)	
RLQ Pain	Yes	308(91.4%)	41(65.1%)	0.3
	No	29(8.6%)	22(34.9%)	
Rebound tenderness	Yes	195(57.9%)	19(30.2%)	<0.001
	No	142(42.1%)	44(69.8%)	
Fever	Yes	166(49.3%)	27(42.9%)	0.4
	No	171(50.7%)	36(57.1%)	
Leukocytosis	Yes	301(89.3%)	26(41.3%)	0.1
	No	36(10.7%)	37(58.7%)	

**TABLE 3:** Sensitivity, specificity, PPV, and NPV of Alvarado score components

	Sensitivity (%)	Specificity (%)	PPV (%)	NPV (%)
Migratory right iliac fossa pain	93.5	28.7	46.4	23.8
Anorexia	88.5	25.8	73.5	50.7
Nausea and vomiting	88.1	32.1	84.2	11.2
RLQ tenderness	88.2	43.1	91.3	65.1
Rebound tenderness	91.1	23.6	57.8	30.1
Fever	85.9	17.3	49.2	42.8
Leukocytosis	92.1	50.6	89.3	41.2
MAS	91.3	38.4	87.7	51.2

PPV=positive predictive value; NPV=negative predictive value; MAS=modified Alvarado score system

**TABLE 4:** Distribution of modified Alvarado scores among children

Alvarado score	Appendicitis(+)	Appendicitis(-)
1-4	3(0.9%)	45(71.4%)
5-6	104(30.9%)	18(28.6%)
7-9	230(68.2%)	0

## DISCUSSION

In the current study, sensitivity of MAS was 91.3%. In the study by Khanafer et al., sensitivity of MAS was 83.3%^6^. In the study by Macklin et al, sensitivity of modified Alvarado score ≥7 was 76.3^7^. In another study from India in adult patients, MAS was sensitive but with a relatively low specificity^9^. Specificity of MAS was 38.4 in the current study which is slightly higher than Khanafer et al. study^6^. Specificity of MAS in Macklin et al study was 78.8%. Macklin et al.^7^ calculated specificity for MAS ≥7 but we calculated overall specificity. 

PPV of MAS in our study was 87.7% which is significantly higher 36.0% in Khanafer et al. study^6^. NPV was 51.2% in our report which was significantly lower than 83.6% in Khanafer et al. study^6^.

In the current study, all children with MAS ≥7, had appendicitis according to pathology report. In our study, 100% of children scored >7 had positive appendicitis. In the study by Kanumba et al.^5^, among children with MAS ≥7, 97.3% had appendicitis which is slightly lower than our study. This difference may be due to age of the patients. 

## CONCLUSION

Modified Alvarado score has high sensitivity but low specificity for diagnosis of acute appendicitis in children.
